# Characterization of Genetic and Allelic Diversity Amongst Cultivated and Wild Lentil Accessions for Germplasm Enhancement

**DOI:** 10.3389/fgene.2020.00546

**Published:** 2020-06-10

**Authors:** Ruwani Dissanayake, Shivraj Braich, Noel O. I. Cogan, Kevin Smith, Sukhjiwan Kaur

**Affiliations:** ^1^Agriculture Victoria, AgriBio, Centre for AgriBioscience, Bundoora, VIC, Australia; ^2^Faculty of Veterinary and Agricultural Sciences, The University of Melbourne, Parkville, VIC, Australia; ^3^School of Applied Systems Biology, La Trobe University, Melbourne, VIC, Australia; ^4^Agriculture Victoria, Hamilton, VIC, Australia

**Keywords:** pulse, SNP markers, allele frequency, GenBank, sequence variant, gene annotation, introgression

## Abstract

Intensive breeding of cultivated lentil has resulted in a relatively narrow genetic base, which limits the options to increase crop productivity through selection. Assessment of genetic diversity in the wild gene pool of lentil, as well as characterization of useful and novel alleles/genes that can be introgressed into elite germplasm, presents new opportunities and pathways for germplasm enhancement, followed by successful crop improvement. In the current study, a lentil collection consisting of 467 wild and cultivated accessions that originated from 10 diverse geographical regions was assessed, to understand genetic relationships among different lentil species/subspecies. A total of 422,101 high-confidence SNP markers were identified against the reference lentil genome (cv. CDC Redberry). Phylogenetic analysis clustered the germplasm collection into four groups, namely, *Lens culinaris*/*Lens orientalis*, *Lens lamottei*/*Lens odemensis*, *Lens ervoides*, and *Lens nigricans*. A weak correlation was observed between geographical origin and genetic relationship, except for some accessions of *L*. *culinaris* and *L*. *ervoides*. Genetic distance matrices revealed a comparable level of variation within the gene pools of *L*. *culinaris* (Nei’s coefficient 0.01468–0.71163), *L*. *ervoides* (Nei’s coefficient 0.01807–0.71877), and *L*. *nigricans* (Nei’s coefficient 0.02188–1.2219). In order to understand any genic differences at species/subspecies level, allele frequencies were calculated from a subset of 263 lentil accessions. Among all cultivated and wild lentil species, *L*. *nigricans* exhibited the greatest allelic differentiation across the genome compared to all other species/subspecies. Major differences were observed on six genomic regions with the largest being on Chromosome 1 (c. 1 Mbp). These results indicate that *L. nigricans* is the most distantly related to *L. culinaris* and additional structural variations are likely to be identified from genome sequencing studies. This would provide further insights into evolutionary relationships between cultivated and wild lentil germplasm, for germplasm improvement and introgression.

## Introduction

The genus *Lens* is a self-pollinating, diploid (2n = 2x = 14), cool season legume with a large genome size of c. 4 Gbp (Singh et al., 2018). With respect to the global production, cultivated lentil is ranked third after chickpea and pea^1^. The origin of lentil comes from the Eastern Mediterranean region, during the Neolithic age around 7000–10,000 years ago (Gupta et al., 2011). Lentil and its wild relatives are naturally distributed throughout the world especially in southwest Asia and Mediterranean regions and were later introduced into North and South America and Australia (Hamwieh et al., 2009). Lentil is produced in over 58 countries, with Canada being the largest producer^1^, generating 48.1% of the world’s production, as well as being the world’s largest exporter accounting for 64.0% of global lentil exports^2^. Despite being the world’s second largest producer (15.7%), India is still the world’s largest importer of lentil^1,2^.

Genetic diversity is the primary basis for crop enhancement and successful plant breeding. Discovery and characterization of novel genes/alleles that can be introgressed into elite germplasm offers opportunities for genetic improvement (Govindaraj et al., 2014; Khazaei et al., 2016). Crosses made between highly divergent parents can be the most valuable for improvement in agronomic characteristics and higher productivity (Fu et al., 2014). Therefore, it is essential to well characterize the lentil gene pool in order to make sustainable gains in crop productivity. Significant levels of genetic diversity have been reported in the genus *Lens* for a range of agronomic and phenological traits (e.g., Gupta and Sharma, 2006; Cristóbal et al., 2014). A total of 58,405 *Lens* accessions are being held in genebanks worldwide (FAO, 2010), with 18.6% residing at the International Center for Agricultural Research in the Dry Areas (ICARDA), Lebanon, while the remainder are held across 103 countries (FAO, 2010). In Australia, numerous lentil accessions (5251 accessions; 9% of the worldwide collection) collected from different geographical regions of the world are preserved within the Australian Grains Genebank (AGG), Horsham, VIC, Australia. The AGG lentil collection is composed of wild species (4.0%), landraces/old cultivars (54.0%), research materials/breeding lines (10.0%), advanced cultivars (5.0%), and other types (26.0%), which are unknown or a mixture of two or more types.

Assessments of genetic diversity and relationships among these preserved germplasms is an important task for facilitating reliable documentation of genetic resources (Govindaraj et al., 2014; Lombardi et al., 2014), which can then be used for selecting relevant material for crop improvement. This phenomenon is particularly valuable to broaden diversity in cultivated lentil, where domestication has reduced levels of resistance to biotic and abiotic stresses compared to its wild relatives (Wong et al., 2015; Malhotra et al., 2019). Attempts have been made to introgress desirable characters of wild lentil taxa into cultivated varieties (Singh et al., 2018). However, the cross-compatibilities and taxonomic relationships between crops and their wild relatives influences the fertility of the final progeny (Singh et al., 2018). For lentil, the cross-ability of cultivated lentil with species of primary (*L*. *culinaris*, *L*. *orientalis*, and *Lens tomentosus*) and secondary (*L*. *odemensis*, *L*. *lamottei*) gene pools was found to be higher compared to the species from tertiary (*L*. *ervoides*) and quaternary (*L*. *nigricans*) gene pools (Gupta et al., 2011). Among the successful crosses made between primary gene pools, the fertility of hybrids varies with the final chromosome pairing and chromosome arrangement within themselves (Ladizinsky, 1979; Singh et al., 2018; Chahota et al., 2019).

Genetic diversity in both wild and cultivated lentil has already been explored using several approaches (Poyraz, 2016). Several studies have used DNA-based markers to evaluate different sets of diverse germplasm for understanding levels of genetic diversity in lentil (Ferguson et al., 1998; Fikiru et al., 2007; Lombardi et al., 2014; Idrissi et al., 2015; Wong et al., 2015; Khazaei et al., 2016; Yadav et al., 2016). Among different marker systems, single base variants (SNPs) are the most abundant molecular marker type present within a genome (Agarwal et al., 2008). The identification of SNPs using high-throughput sequencing technology is known as genotyping-by-sequencing (GBS) (Chung et al., 2017). Since the development of next-generation sequencing (NGS) platforms has continuously driven the cost of sequencing down, GBS methods are now practicable for highly diverse and large genome species (Malmberg et al., 2018a). The main features of GBS include low cost, ease of scaling sample numbers, ability to identify and genotype large numbers of SNPs, reduced sample handling, few amplification and clean up steps, and efficient barcoding compared to other genotyping methodologies. Therefore, GBS methods have become popular as a cost-effective and unique tool for genomic assisted breeding in plant species (Chung et al., 2017). GBS methods can be divided into target enrichment/capture-based methods and genome complexity reduction-based methods (Malmberg et al., 2018a). Among them, the transcriptome-based complexity reduction approach is the most reliable method that allows the detection of sequence polymorphisms and splice variants (Sudheesh et al., 2016; Malmberg et al., 2018a). The protocol has successfully been used to genotype a number of crop species including alfalfa (Yang et al., 2011), maize (Hansey et al., 2012), wheat (Ramirez-Gonzalez et al., 2015), and legume crops such as chickpea (Hiremath et al., 2011) and lentil (Malmberg et al., 2018a).

Various data analysis pipelines and software packages have also been developed in line with advancements in NGS platforms to align, map, and identify variants. Burrows wheeler alignment tools such as BWA (Li and Durbin, 2010) and Bowtie (Langmead, 2010) were developed to align NGS short read data. With time, TopHat2 and STAR (Spliced Transcripts Alignment to a Reference) aligner mapping tools were developed for aligning RNA sequencing (RNA-seq) data to the reference genome to identify exon–exon splice junctions (Kim et al., 2013; Dobin and Gingeras, 2016). Many bioinformatic pipelines such as SAMtools (Li et al., 2009), Freebayes (Garrison and Marth, 2012), Atlas2 (Challis et al., 2012), GATK (Van der Auwera et al., 2013), and glfTools^3^ have also been developed for variant calling of NGS data. Among them, SAMtools-mpileup and GATK-Haplotype Caller (GATK-HC) are widely used variant calling programs to accurately and consistently identify genome variants (Warden et al., 2014; Clevenger et al., 2015).

So far, only a limited number of SNP-based genetic diversity studies performed on global lentil collections comprising both wild and cultivated lentil accessions have been published (Lombardi et al., 2014; Wong et al., 2015; Khazaei et al., 2016). The present study was focused on the assessment of genetic diversity of a group of 467 lentil accessions, collected from diverse geographical origins using SNP markers. The transcriptome-based complexity reduction method was used for genotyping. Different bioinformatic pipelines were tested to generate a set of high-confidence SNP markers in order to assess genetic diversity among cultivated and wild lentil accessions. Allele frequency-based analysis was also performed to understand allelic variation among cultivated and wild lentil species/subspecies. The outputs of this study will provide a strong basis for future lentil genetic and genomic studies as well as assist lentil breeders to make informed selections.

## Materials and Methods

### Plant Materials

Seeds of 467 lentil accessions, including wild (*L. orientalis*, *L. lamottei*, *L. odemensis*, *L*. *ervoides*, and *L. nigricans* – 163) and cultivated species (*L. culinaris* – 304) were obtained from the AGG, Horsham, VIC, Australia. The geographical origins of these accessions varied widely with the majority being from Australia and the Middle East. The remaining accessions were derived from North and South America, Africa, Mediterranean, and European and Asian countries, with the number of accessions from each geographical origin varying from 2 to 240 (Table 1). To obtain leaves for RNA extraction, two to three seeds of each accession were germinated for 2–3 weeks in glasshouse at 22 ± 2°C under a 16-h/8-h (light/dark) photoperiod, in individual pots filled with standard potting mix at the premises of Agriculture Victoria, AgriBio Centre, Bundoora, VIC, Australia. Fresh leaf tissues were sampled from single seedlings and immediately frozen in liquid nitrogen before storage at −80°C.

**TABLE 1:** Geographical details of the lentil accessions used in this study.

	Country	Geographical origin	No. of accessions
1	Canada, United States	N America	11
2	Chile, Argentina	S America	4
3	Ethiopia	Africa	2
4	Tunisia, Spain, France, Italy, Croatia, Bosnia and Herzegovina	Mediterranean	25
5	Czechia, Yugoslavia	Central Europe	14
6	Cyprus, Turkey, Syria, Jordan, Israel, Iran	Middle East Asia	125
7	Russia, Armenia, Azerbaijan	Eurasia	10
8	Uzbekistan, Tajikistan	Central Asia	3
9	Afghanistan, India	South Asia	4
10	Australia	Australia	240

### Total RNA Extraction and Library Preparation

Total RNA extraction was performed using the RNeasy 96 Plant Kit (QIAGEN, Hilden, Germany) following the manufacturer’s instructions. Using a NanoDrop UV-Visible spectrophotometer (Thermo-Scientific, Wilmington, DE, United States), the concentration and quality of RNA was confirmed at the wavelength ratios of A260/230 and A260/280 nm. The integrity of extracted RNA samples was evaluated using TapeStation 2200 platform with RNA ScreenTape System (Agilent Technologies, Santa Clara, CA, United States), following the manufacturer’s guidelines. RNA-Seq libraries were prepared using the Agilent SureSelect Strand Specific RNA Library Preparation Kit and evaluated on TapeStation 2200 platform with D1000 ScreenTape System (Agilent Technologies, Santa Clara, CA, United States). Each RNA-seq library was paired-end (2 × 151 bp) sequenced using Illumina Hiseq 3000 Sequencing platforms (Illumina Inc., San Diego, CA, United States).

### Read Mapping

Following fastq data generation, the raw sequence reads were filtered using a custom perl script to remove adaptor sequences along with reads and bases of low quality (*Q* ≤ 30). Reads with three consecutive unassigned nucleotides (N) were also trimmed and finally any reads shorter than 50 bp in length were removed from the final set. The remaining high-quality trimmed sequence reads were aligned to the lentil reference genome sequence of cultivar CDC Redberry (version 1.2)^4^ using two alignment pipelines, TopHat2 (version 2.1.1) (Trapnell et al., 2009) and STAR aligner (version 2.5.4a) (Dobin et al., 2013). The number of properly paired reads were obtained using the SAMtools flagstat option and comparison of the number and percentage of mapping reads obtained for each method was made (Li et al., 2009).

### Variant Calling and Filtering

**FIGURE 1:**
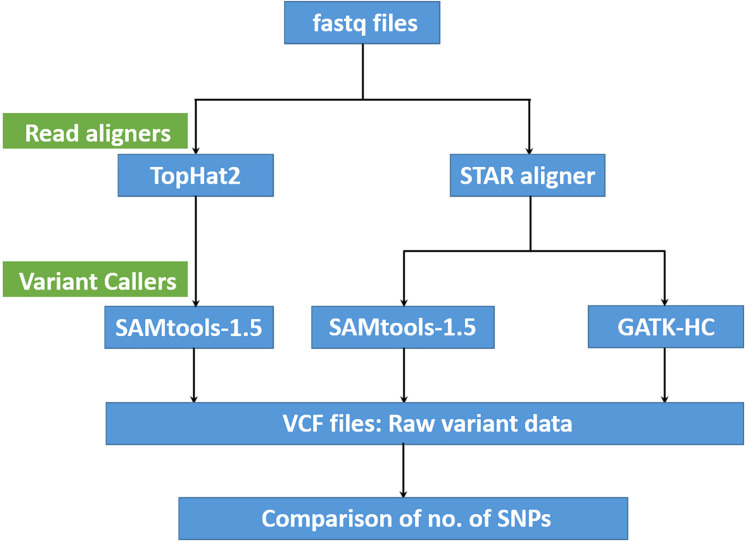
A flow diagram summarizing the performance comparison of variant calling pipelines.

Variant calling was performed using SAMtools (version-1.5) (Li et al., 2009) and GATK-HC (Van der Auwera et al., 2013). Since the number of lentil accessions per species/subspecies was unevenly distributed (*Lens* sp. – 2, *L. lamottei* – 1, *L. odemensis* – 22, *L. nigricans* – 24, *L. ervoides* – 57, *L. orientalis* – 57, and *L. culinaris* – 304), variant calling was performed separately for each species. The number of SNPs obtained from each of the mapping and variant calling methods were compared to identify the best analysis pipeline (Figure 1). The STAR aligner-GATK-HC pipeline was chosen as the best mapping and variant calling option. A unique SNP list was created by combining and sorting the individual lists that were generated from each species. Final variant calling was then run on all samples using the before mentioned SNP list using SAMtools to generate a combined final output in VCF format. The final VCF output was then filtered based on various parameters: depth (DP ≥ 5), maximum allelic frequency (MAF = 0.05), maximum missing data (50%), and base quality (Q30).

### Phylogenetic Tree Construction and Structure Analysis

Genetic diversity analysis was performed using the abovementioned filtered SNP data from all accessions. Genetic distances for each lentil accession were calculated using Nei’s method within the StAMPP package (Pembleton et al., 2013). A phylogenetic tree was constructed using the unweighted neighbor-joining (NJ) method, as implemented in the DARwin-6.0.17 software (Perrier and Jacquemoud-Collet, 2006). The relationship between different lentil accessions was evaluated based on their geographical origin as listed in Table 1, and analysis of population structure was performed using fastSTRUCTURE (version 1.0) (Raj et al., 2014). At least 20 runs of STRUCTURE were performed by setting K from 1 to 20. Finally, the chooseK option in fastSTRUCTURE was used in identifying the marginal likelihood value to select model components for admixture model.

### Allele Frequency-Based Diversity Analysis

A subset of 263 lentil accessions (*L. odemensis* – 22, *L. nigricans* – 24, *L. ervoides* – 57, *L. orientalis* – 57, and *L. culinaris* – 103) was chosen based on the sample numbers that belonged to each species/subspecies to capture a comparable representation from each group. Variant calling and filtering were again performed using pipelines and parameters discussed above, to generate the final output in VCF format. The allele frequencies for each SNP position were calculated using VCFtools-freq output option (Danecek et al., 2011). Only the allele frequencies from alternate alleles were considered in analysis. Alternate allele frequency differences were calculated with respect to *L*. *culinaris*, and the dataset was evaluated to identify the unique genomic regions that varied among different lentil species/subspecies. The outcomes were interpreted using allele plots, heatmaps, and graphical representations. The respective heatmaps were generated using the software package R Studio with the ComplexHeatmap function from the CRAN library (R Core Team, 2018).

### Gene Annotation

Regions of genomic variation among different species/subspecies were assessed for intron/exon boundaries using the gene-finding format (GFF) file from CDC Redberry. Based on the exon–intron positions specified in the GFF file, the sequences from the corresponding genes were extracted from the CDC Redberry genome (version 1.2) fasta file using BEDtools (version 2.26.0) (Quinlan and Hall, 2010). The extracted fasta sequences were also BLAST analyzed in NCBI-Basic Local Alignment Search Tool, nucleotide (BLASTN 2.8.1+) option to obtain corresponding gene annotations.

## Results

### SNP Identification and Filtration for Genetic Diversity Analysis

The total number of SNPs or variants identified for all species/subspecies was 3- to 10-fold higher when STAR aligner was used, as compared to TopHat2 (Table 2), which is also irrespective to the subsequent variant identification pipeline used. Comparing the output that used STAR aligner, the GATK-HC variant caller was found to be optimal as it identified a marginally higher number of SNPs in total as well as at the species/subspecies levels.

**TABLE 2:** Number of SNPs identified through different data analysis pipelines.

		SNP calling methods (without filtering)		
Lentil accessions	No. of accessions	TopHat2-SAMtools	STAR aligner-SAMtools	STAR aligner-GATK-HC
***Lens* sp.**	2	23,007	255,235	316,260
***L. lamottei***	1	59,506	552,980	599,561
***L. odemensis***	22	556,480	3,056,657	3,203,316
***L. nigricans***	24	565,066	3,679,329	3,607,883
***L. ervoides***	57	1,149,872	5,859,835	5,540,000
***L. orientalis***	57	1,416,431	5,831,877	5,638,279
***L. culinaris***	304	4,429,865	5,793,641	8,243,433
	**Unique SNPs identified**	6,514,072	15,592,178	18,045,718

A total of 18,045,718 SNPs identified from STAR aligner-GATK-HC pipeline were first filtered based on sequence depth (DP ≥ 5; otherwise, the SNP was excluded), leaving a set of 12,522,726 SNPs. This subset of SNP markers was further filtered for percentage of missing data (50%), quality score (*Q* ≥ 30; 1,414,996 SNPs), and minor allele frequencies (MAF = 0.05) that resulted in a final set of 422,101 high-quality SNP markers (Supplementary Table S1), to be used in phylogenetic analysis (Table 3).

**TABLE 3:** Number of SNPs identified in genetic diversity and allele frequency-based genomic analysis.

	Phylogenetic tree construction	Allele-frequency-based analysis
	
Filtering step	No. of SNPs remaining after each filtering step	
DP 5	12,522,726	11,099,596
Maximum missing 50% and Q30	1,414,996	1,345,992
MAF 0.05	422,101	563,692

### Phylogenetic Tree Construction

**FIGURE 2:**
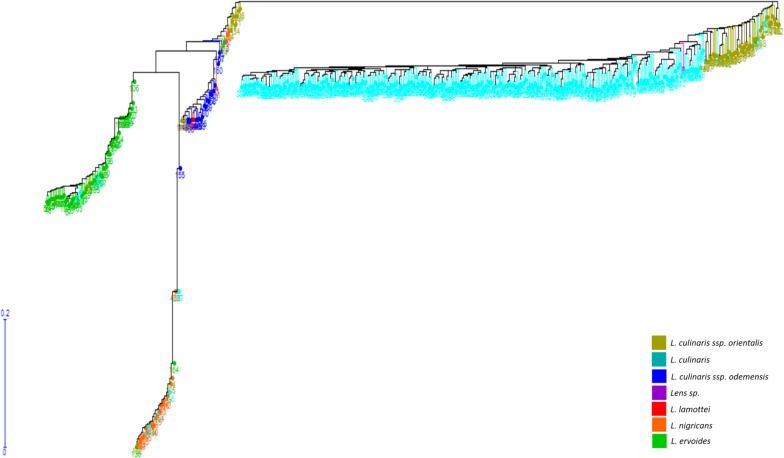
Unweighted neighbor joining dendrogram generated based on genetic distance calculation from StAMPP in R.

Genetic distance calculations were performed among 467 lentil accessions (Supplementary Table S2). Genetic diversity was assessed using the unweighted NJ dendrogram that classified the diverse lentil germplasm into four major groups, namely, *L*. *culinaris*/*L*. *orientalis*, *L*. *lamottei*/*L*. *odemensis*, *L*. *ervoides*, and *L*. *nigricans* (Figure 2). Among these species, *L*. *orientalis* was found to be closest to the *L*. *culinaris*, followed by *L*. *odemensis*/*L*. *lamottei*, *L*. *ervoides*, and *L*. *nigricans*. As shown in Figure 2, *L*. *orientalis* was further sub-divided into two clusters, where one was closer to *L*. *culinaris* and the other clustered closer to *L*. *odemensis*. A single genotype (ILWL437) that was originally classified as *L*. *lamottei* actually clustered within *L*. *odemensis*. In this study, the two lentil accessions (32601 and BGRC-025688), classified as *Lens* sp., were clustered in *L. culinaris* and *L*. *orientalis* gene pools, respectively. Moreover, 22 accessions out of 467 lentil accessions were classified in clusters that did not correlate to the passport data obtained from AGG. This was more visible in *L*. *orientalis*, *L*. *odemensis*, *L*. *ervoides*, and *L*. *nigricans* clusters (Figure 2). Even though the germplasm set contained high proportion of *L. culinaris* accessions (304) with majority being originated from Australia, there was no bias observed in terms of clustering. This was further confirmed by regenerating the phylogenetic tree after removing 201 of the 304 *L. culinaris* accessions (retaining the key ancestral accessions plus released varieties) (Supplementary Figure S1).

Based on genetic distance calculations, the most divergent pair was observed between *L*. *ervoides* (ILWL042) and *L*. culinaris (10H063L-12HS3003) accessions with Nei’s coefficient value of 0.92295. The two *L*. *orientalis* accessions ILWL211 and ILWL069, both originating from the Former Soviet Union, exhibited the lowest genetic distance (Nei’s coefficient value 0.0043). Further analysis was performed to understand the variation in genetic distances between and within lentil species/subspecies (Table 4). *L*. *culinaris* exhibited higher genetic distance to *L*. *ervoides* (0.33642–0.89817) and *L*. *nigricans* (0.31141–0.89375) compared to *L. orientalis* (0.12622–0.13927) and *L*. *odemensis* (0.27913–0.31805). Further analysis revealed wide genetic distance variations within the species/subspecies level [*L*. *culinaris* (0.01468–0.71163), *L*. *ervoides* (0.01807–0.71877), and *L*. *nigricans* (0.02188–1.2219)].

**TABLE 4:** Ranges of Nei’s coefficient values within and amongst different lentil species/subspecies.

	*L*. *culinaris*	*L. ervoides*	*L. nigricans*	*L. orientalis*	*L. odemensis*
***L*. *culinaris***	0.01468–0.71163	0.33642–0.89817	0.31141–0.89375	0.12622–0.13927	0.27913–0.31805
***L*. *ervoides***		0.01807–0.71877	0.25233–0.82594	0.13088–0.42042	0.16502–0.79800
***L*. *nigricans***			0.02188–1.2219	0.20977–0.83887	0.26083–0.68011
***L*. *orientalis***				0.00646–0.5607	0.11997–0.28186
***L*. *odemensis***					0.01826–0.34535

Within *L*. *culinaris*, some lentil accessions originating from Canada (1894T, Indianhead) showed wide genetic distances to some of the Australian (CIPAL0413, PBA Flash, Cobber) and Middle East Asian accessions (ILL5714, ILL6802, Northfield, Commando), with Nei’s coefficient value between 0.60 and 0.70. Similar results were also observed within *L*. *ervoides*, where distant genetic relationships were observed within two Mediterranean lentil accessions ILWL042 and HOFFMAN-42 (Nei’s coefficient value 0.7178). ILWL042 also showed wide genetic distances to multiple lentil accessions originated from Middle East Asia, Russia, and Central Europe (Supplementary Table S3). Similarly, within *L*. *nigricans*, HOFFMAN-22, HOFFMAN-32, and ILWL014 showed distant genetic relationship to many Mediterranean, Middle East Asian, Central Asian, and Central European lentil accessions with genetic distances of up to 1.10 (Supplementary Table S3).

**FIGURE 3:**
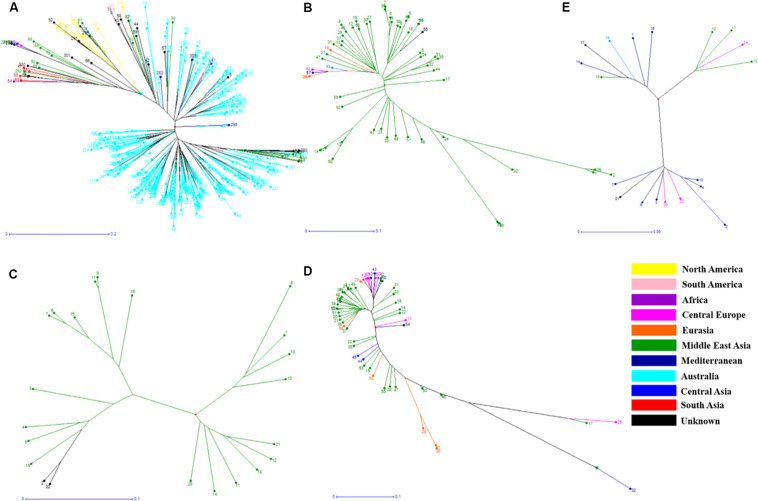
Phylogenetic trees from different Lens species based on their geographical origin. **(A)** *L*. *culinaris*, **(B)** *L*. *orientalis*, **(C)** *L*. *odemensis*, **(D)** *L*. *ervoides*, and **(E)** *L*. *nigricans*. The accessions are color coded according to the details listed in Supplementary Table S2.

**FIGURE 4:**
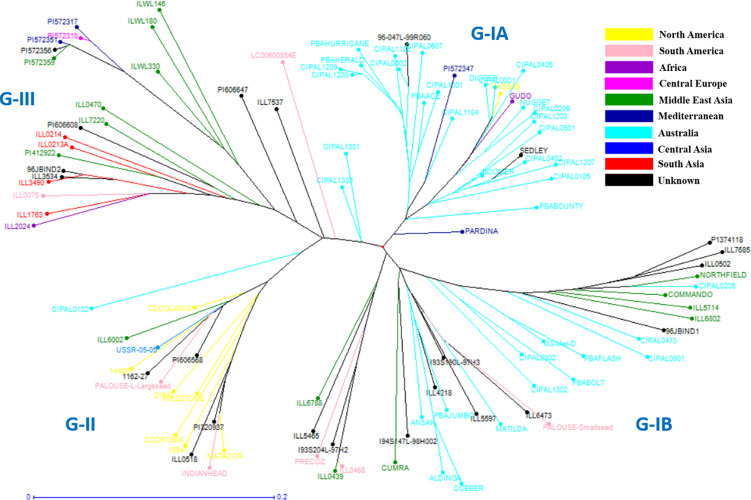
Detailed unweighted neighbor joining dendrogram generated on key *L*. *culinaris* accessions.

In order to understand the genetic relationships between lentil genotypes with respect to their geographical origin, lentil accessions were evaluated on this basis (Supplementary Table S2). However, a weak correlation was observed between geographic origins and lentil species/subspecies especially in *L*. *orientalis*, *L*. *odemensis*, and *L*. *nigricans* (Figure 3 and Supplementary Figure S2). Some of the lentil accessions from *L*. *culinaris* and *L*. *ervoides* did, however, show a degree of relationship to their geographical origin. As shown in Figure 3D, some of the Central European *L*. *ervoides* accessions (e.g., HOFFMAN-37, 38, 39, 41, and 45) were clustered separately from the Middle East Asian *L*. *ervoides* accessions (e.g., ILWL-129, 262, 263, HOFFMAN-99, 100, 102, etc.). *L*. *culinaris* lines from Australia, North America, Asia, and Mediterranean regions were also clustered separately (Figures 3A, 4). The *L*. *culinaris* dendrogram in Figure 4 identified four major groups (G-IA, G-IB, G-II, and G-III). Group-IA and IB mainly consisted of Australian germplasm (e.g., PBA Herald, PBA Hurricane, PBA Ace, Cassab and CIPAL 1203, PBA Flash, PBA Bolt, PBA Jumbo), whereas some South American lentil accessions (e.g., PRECOZ, ILL0468, and ILL0439) were clustered as a small group, separately from Australian lines in Group-IB. Most of the lentil accessions originated from North America (e.g., CDC Glaims, CDC Matador, CDC Robin, and Milestone) and Asia (e.g., ILL0214, ILL0213, ILL1763, and ILL3490) were clustered into Group II and Group III, respectively (Supplementary Table S2).

### Population Structure Analysis

Structure analysis revealed an optimum value of *K* = 5, suggesting five distinct clusters for the cultivated and wild lentil germplasm (Supplementary Figure S3), namely, *L. culinaris*, *L. orientalis*, *L. odemensis*, *L. ervoides*, and *L. nigricans* populations. Further analysis revealed that both *L. odemensis* and *L. lamottei* belonged to same cluster while accessions originally named as *Lens* sp. were grouped under *L. orientalis* (Supplementary Figure S3) cluster. Although, *L. culinaris* and *L. orientalis* populations were categorized into two clusters, substantial overlaps were observed (indicated in blue and red color, Supplementary Figure S3).

### Allele Frequency-Based Diversity Analysis

A total of 563,692 high-quality polymorphic SNP markers were identified from a subset of 263 accessions for use in allele frequency-based analysis (Table 3 and Supplementary Table S4). However, the final analysis was performed on only 490,205 SNP positions that covered the seven pseudomolecules in the reference genome (v1.2) of CDC Redberry (Chromosome 1: 74,000, Chromosome 2: 80,234, Chromosome 3: 68,440, Chromosome 4: 71,745, Chromosome 5: 74,863, Chromosome 6: 56,204, and Chromosome 7: 64,719), ignoring the SNPs that were identified on unanchored scaffolds and contigs.

**FIGURE 5:**
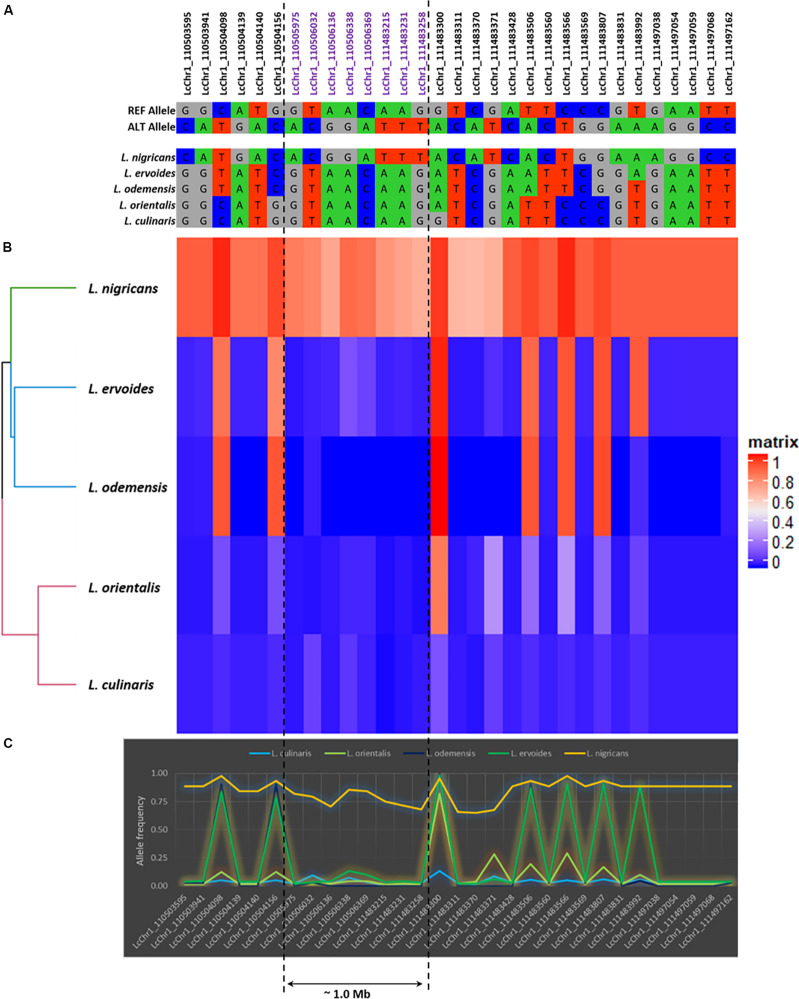
The detailed illustration of the genetic polymorphism detected in LcChr1. **(A)** Allelic polymorphism. **(B)** Heatmap based on allele frequencies (in matrix, 1: Alternative allele and 0: Reference allele). **(C)** Graphical representation of the allele frequencies.

Based on allele frequencies, *L*. *nigricans* exhibited the greatest allelic differentiation as compared to the other four species (Figure 5 and Supplementary Figure S4), having higher frequency of alternate allele across all chromosomes. This was further confirmed from the results of the genetic diversity analysis (Figure 2), where *L*. *nigricans* showed the maximum genetic distance to *L*. *culinaris*. A large number of allelic variations were observed across the genome; however, most of them spanned very small physical distances (10–400 bp). In order to explore major variations at the exome levels, we focused on regions with physical distance greater than 1.0 kb (Supplementary Table S5). Six regions were identified on Chromosome 1 (Region 1: LcChr1_110505975-111483258; 1.0 Mb), Chromosome 2 (Region 2: LcChr2_291843749-291844813; ∼1 kb), Chromosome 4 (Region 3: LcChr4_27744411-27753914; 9.5 kb and Region 4: LcChr4_220029017-220032998; ∼3.9 kb), and Chromosome 7 (Region 5: LcChr7_35746842-35770194; ∼23.0 kb and Region 6: LcChr7_35770207-35773931; ∼3.7 kb). Among them, *L*. *nigricans* accounted for the allelic differentiation on five of the six regions, while *L. ervoides* differed on Region 4 in comparison to the other species (Supplementary Figure S4C). To further analyze the divergence in the lentil genome across species/subspecies, allele frequency variations from the abovementioned six genomic regions were analyzed in detail and illustrated in Figure 5 and Supplementary Figure S4.

Potential candidate genes associated with variable genomic regions were identified using the GFF file from CDC Redberry genome as well as BLASTN analysis in NCBI (Table 5). A total of five candidate genes of varying annotations were identified on Region 1 (LcChr1_110505975-111483258; 1.0 Mb), including transcription initiation factor TFIID (LcChr1_110507918-110511568), importin subunit alpha (LcChr1_110758323-110760021), copper transporter (LcChr1_111384460-111384924), cellular nucleic acid-binding protein (LcChr1_111461822-111462649), and polyglutamine-binding protein/nuclear cap-binding protein subunit (LcChr1_111483114-111510662). However, no candidate genes from Region 2 (LcChr2_291843749-291844813; ∼1 kb) and Region 6 (LcChr7_35770207-35773931; ∼3.7 kb) were identified, due to their narrow physical distances. A BLASTN similarity search of these six regions to other plant species revealed high-confident matches (ranging from 70 to 87%) to legume species, *Cajanus cajan* (pigeon pea) and *Pisum sativum* (field pea). Potential candidate genes identified are summarized in Table 5.

**TABLE 5:** Genes identified for the major chromosome regions.

Chromosome regions	Start	End	Gene information gained from CDC Redberry (version 1.2) gff file	BLASTN results			SNP Markers
				GenBank Accession	Identity	Description	
LcChr1 (110505975–111483258)	110507918	110511568	ID = Lc01774; Name = Lc01774; Description = “Transcription initiation factor TFIID, subunit TAF11”	XM_013611759.2	83.0%	PREDICTED: *Medicago truncatula* transcription initiation factor TFIID subunit 11 (LOC25483108), mRNA	×
	110758323	110760021	ID = Lc02039; Name = Lc02039; Description = “Importin subunit alpha”	XM_024771107.1	73.0%	PREDICTED: *Medicago truncatula* importin subunit alpha-4 (LOC25499660), mRNA	×
				XM_012715998.1	75.0%	PREDICTED: *Cicer arietinum* importin subunit alpha-4-like (LOC101508144), transcript variant X2, mRNA	×
	111384460	111384924	ID = Lc04312; Name = Lc04312; Description = “Uncharacterized protein”	XM_013600715.2	82.0%	PREDICTED: *Medicago truncatula* copper transporter 6 (LOC25492554), mRNA	×
				XM_004507092.3	83.0%	PREDICTED: *Cicer arietinum* copper transporter 6-like (LOC101509977), mRNA	×
	111461822	111462649	ID = Lc04887; Name = Lc04887; Description = “Cellular nucleic acid-binding protein”	AF155746.1	86.0%	*Pisum sativum* clone Psat6 repetitive sequence	×
	111483114	111510662	ID = Lc04888; Name = Lc04888; Description = “Polyglutamine-binding protein”	XM_020363131.1	70.0%	PREDICTED: *Cajanus cajan* nuclear cap-binding protein subunit 1 (LOC109801952), transcript variant X3, mRNA	√
LcChr4 (27744411–27753914)	27736771	27757110	ID = Lc16633; Name = Lc16633; Description = “UDP-glucose:sterol 3-O-glucosyltransferase”	XM_013602437.2	87.0%	PREDICTED: *Medicago truncatula* ABC transporter A family member 7 (LOC25493811), mRNA	√
LcChr4 (220029017–220032998)	220028589	220033612	ID = Lc18023; Name = Lc18023; Description = “Mitogen-activated kinase kinase kinase alpha”	XM_003630226.3	81.0%	PREDICTED: *Medicago truncatula* mitogen-activated protein kinase kinase kinase 3 (LOC11436239), mRNA	√
LcChr7 (35746842–35770194)	35769951	35774027	ID = Lc31767; Name = Lc31767; Description = “Uncharacterized protein”	DQ070849.1	81.0%	*Glycine max* SjR2-like retrotransposon reverse transcriptase family member gene, complete cds	√

## Discussion

### Assessment of Genetic Diversity and Population Structure in Lentil Germplasm

The current study has identified taxonomic relationships among cultivated and wild lentil gene pools using SNPs identified from transcriptome sequencing. After comparing different SNP detection pipelines, the STAR aligner-GATK-HC pipeline was chosen as the preferred method for SNP calling due to higher number of SNPs detected compared to other pipelines tested. Similar results have been reported in other studies where the efficiency of SNP and indel detection from both whole genome and exome-captured sequence data was found to be higher in GATK-HC as compared to SAMtools-mpileup (Yu and Sun, 2013; Warden et al., 2014; Poplin et al., 2017). To accurately capture the information at each species/subspecies level, the variant calling was performed separately for each species/subspecies. Thus, this study has delivered an enriched SNP resource to be used by breeders to make informed selections in breeding programs.

Genetic diversity analysis clustered the six *Lens* species into four major groups, *L*. *culinaris*/*L*. *orientalis*, *L*. *odemensis*/*L*. *lamottei*, *L*. *ervoides*, and *L*. *nigricans*, which is consistent with results from Wong et al. (2015). However, structure analysis categorized *L*. *orientalis* and *L*. *culinaris* separately, which is not consistent with the results of Wong et al. (2015). The divergence in the findings between the two studies may be due to the difference in the number of accessions studied (80 accessions) as well as number of SNP markers (c. 5000) used to perform the analysis. Some of the lentil accessions used in this study were clustered outside of their known identification based on genebank passport information. The inconsistencies highlight the importance of the application of molecular tools to genebanks to improve the quality of the data that can be accessed.

A weak correlation was observed between clusters and geographical origins, with some exceptions, which is in line with some previous studies (Lombardi et al., 2014; Khazaei et al., 2016). The reason for weak correlation may be due to the fact that most lentil accessions originated from the Mediterranean regions and were later distributed across the globe (Khazaei et al., 2016). The movement of lentil and the weak correlation with geographic origin results in a lower value on traditional genebank passport data for this species, and DNA-derived estimates of relatedness are far more valuable and informative. Genetic relationships among various lentil varieties were confirmed based on pedigree data. For example, CDC-Matador showed a genetic distance of 0.0233 to its parental line Indianhead and was clustered in the same cluster. Similarly, PBA Flash showed genetic distances of 0.0541 and 0.0487 to its parental lines Nugget (Australia) and ILL7685 (unknown, AGG), respectively. Information generated on levels of genetic distance among various lentil species/subspecies provide a great opportunity to select accessions for breeding purposes. It has been demonstrated that two accessions with moderate levels of genetic distance can be easily crossed to introgress traits of interest. For example, both Indianhead and Northfield (syn. ILL5588, Nei’s coefficient value 0.69194), known to be resistant to Ascochyta Blight (Rodda et al., 2017), have been crossed to each other to produce improved levels of disease resistance in the breeding germplasm (Rodda et al., 2017). The information generated in the current study provides a database for breeders to select *Lens* accessions for future crossing strategies in order to introgress novel alleles.

Large genetic distances observed between cultivated and wild lentil accessions (*L*. *ervoides* and *L*. *nigricans*) indicate that direct hybridization between cultivated and wild lentil accessions that belong to tertiary and quaternary gene pools may be challenging. As an alternative, the use of an intermediate relative as a bridge species may be helpful in transferring alleles/genes that belong to distant gene pools. Attempts have been made in *L*. *nigricans* (quaternary gene pool) to transfer resistant genes for ascochyta blight and anthracnose diseases into *L*. *culinaris* (primary gene pool) via *L*. *ervoides* (tertiary gene pool) as a bridge species (Kumar et al., 2014b). In addition to this, exploring the genetics of landraces from *L. culinaris* may also be highly beneficial as most of them are not yet fully adapted to the current environments and carry beneficial alleles. Crosses made with landraces will be less cumbersome and most likely will result in improved resistances and the genetic base without compromising the fertility levels of the progeny. For example, the lentil landraces such as ILWL042 and ILWL37 included in the current study that are known to have a number of disease resistance genes [powdery mildew, rust and Fusarium wilt (Gupta and Sharma, 2006; Kumar et al., 2014b)] can be crossed with elite lentil varieties to improve disease resistance in the breeding germplasm. Successful attempts have been made to cross an early flowering exotic germplasm “Precoz” with some Indian lentil accessions to diversify the indigenous gene pool (Kumar et al., 2014a).

### Allele Frequency-Based Diversity Analysis and Gene Annotation

In addition to the genetic distances, the genomic differences at species level play a major role in obtaining successful hybridization of wild taxa with cultivated varieties (Singh et al., 2018). The allele frequency-based analysis was performed to get an initial insight into and understanding of the genic differences among different species of *Lens* taxa. In most cases, *L*. *nigricans* differed significantly from the other five species/subspecies by having higher proportions of alternate alleles. The detection of Region 1 on LcChr1 with a physical distance of ∼1.0 Mb indicated that *L*. *nigricans* may have undergone significant divergence compared to other *Lens* species. The reason for this phenomenon is not clear, although Ladizinsky (1979) has shown that *L*. *nigricans* has a slightly different karyotype compared to *L*. *culinaris* and *L*. *orientalis* with the largest chromosome being metacentric instead of submetacentric (Cubero, 1984). This indicates that *L. nigricans* may have undergone stronger selection pressure as compared to other species. Various studies in other crop plants (e.g., potato, bread wheat, canola) have reported genomic rearrangements such as substitutions, insertion, and deletions (Tang et al., 2006; Sidhu et al., 2015; Malmberg et al., 2018b). However, as the current study is restricted to an exon-based transcriptomic analysis and did not sequence inter genic regions, it is unable to detect such kind of variations. Further analysis based on skim genome sequencing may facilitate the identification of such structural variations and other chromosome rearrangements within *Lens* species/subspecies.

A total of five candidate genes were identified from Region 1, which is close to the expected average gene frequency in lentil, based on genome size (7.0 genes per Mb) (Kaur et al., 2011). Further detailed analysis will be needed for a full characterization of these genes to understand their function in wild and cultivated lentil species. However, various studies performed in other wild and cultivated crops (e.g., rice, tomato, barley, cotton) have discussed the functionality of some of the genes identified in the current study including metal transporter gene, ABC transporter gene, and mitogen-activated protein kinase 3 (MAPKK 3) gene. According to Ricachenevsky and Sperotto (2016), the wild varieties of *Oryza* sp., (*Oryza rufipogon* and *Oryza glaberrima*) have shown better response to metal (e.g., phosphate, aluminum, and iron) applications, as compared to the cultivated rice (*Oryza sativa*). These wild species have been used for breeding purposes to enhance the metal tolerance in cultivated rice varieties. In addition, Orsi and Tanksley (2009) identified the functionality of the ABC transporter gene, which controls the QTL that determines seed size of wild and cultivated tomatoes. Moreover, the performance of MAPPK 3 gene has been studied in different crop species. Wang et al. (2016) demonstrated that overexpression of MAPPK 3 can regulate the stomatal response, root hair growth, and thereby the drought stress responses in cotton. Several studies on cultivated and wild lentil accessions have reported superior drought tolerance in *L*. *odemensis*, *L*. *ervoides*, and *L*. *nigricans* as compared to *L. culinaris* (Hamdi and Erskine, 1996; Gorim and Vanderberg, 2017), which indicates that wild lentil species may carry a variant allele of the MAPPK 3 gene. Another study performed in barley has explained the influence of MAPKK 3 on seed dormancy and thereby improvement in seed germination and pre-harvest sprouting tolerance (Nakamura et al., 2016). The candidate genes identified from the current study can be better characterized in relation to the abovementioned functionalities to gain further insights into the wild gene pool of lentil.

## Conclusion

Assessment of genetic diversity among a global collection of wild and cultivated lentil accessions was performed using a set of genome-wide distributed SNP markers. The genetic diversity analysis revealed a clear grouping of lentil germplasm into four major clusters/gene pools, *L*. *culinaris*/*L*. *orientalis*, *L*. *odemensis*/*L*. *lamottei*, *L*. *ervoides*, and *L*. *nigricans*. Wild and cultivated lentil accessions showed a weak correlation to their geographical origins. Therefore, the selection of parents for breeding purposes can be better selected by genetic distance boundaries within and between lentil species/subspecies. Allele frequency-based analysis also confirmed that *L*. *nigricans* was distantly related to *L*. *culinaris*. The identified native and exotic lentil accessions with wide genetic distances and specific agronomical traits can be used in widening the Australian lentil germplasm for breeding purposes.

## Data Availability Statement

The datasets generated for this study can be found in NCBI SRA accession PRJNA625627, https://www.ncbi.nlm.nih.gov/sra/PRJNA625627.

## Author Contributions

SB prepared the plant materials and performed the sequencing library preparation. RD performed the data analysis. SK, NC, and KS provided overall project leadership. RD, NC, SK, and KS all assisted in drafting the manuscript. All authors have read and approved the final manuscript.

## Conflict of Interest

The authors declare that the research was conducted in the absence of any commercial or financial relationships that could be construed as a potential conflict of interest.

## Footnotes

1 http://www.fao.org/faostat/en/#data/QC

2 http://www.fao.org/faostat/en/#data/TP

3 http://csg.sph.umich.edu/abecasis/glfTools/

4 http://knowpulse.usask.ca

## Supplementary Material

FIGURE S1 Unweighted neighbor joining dendrogram generated from all Lens species based on genetic distance calculation from StAMPP in R. Of the *L. culinaris* set, only a proportion of key accessions is included.

FIGURE S2 Genetic relationships between lentil genotypes with respect to their geographical origin. **(A)** *L. culinaris* **(B)** *L. orientalis* **(C)** *L. odemensis* **(D)** *L. ervoides* **(E)** *L. nigricans*.

FIGURE S3 Graphical representation of fastSTRUCTURE results (*K* = 5).

FIGURE S4 Allelic polymorphism detected in other chromosome regions. **(A)** Region 2 on LcChr2 **(B)** Region 3 on LcChr4 **(C)** Region 4 on LcChr4 **(D)** Region 5 and 6 on LcChr7.

TABLE S1 List of c. 4 million high-confidence SNPs used in genetic diversity analysis.

TABLE S2 Information of lentil accessions used in this study and their geographical origins.

TABLE S3 Lentil accessions with wide genetic distances, which are found within *L*. *culinaris*, *L*. *ervoides*, and *L*. *nigricans.*

TABLE S4 List of c. 5 million high-confidence SNPs used in allele frequency–based analysis.

TABLE S5 Data sets used in allele frequency-based analysis.
