# Supplementary figures and images for: Characterization of Genetic and Allelic Diversity Amongst Cultivated and Wild Lentil Accessions for Germplasm Enhancement
Source: Front Genet. 2020 Jun 10;11:546. doi: 10.3389/fgene.2020.00546 (PMC7298104; doi:10.3389/fgene.2020.00546)

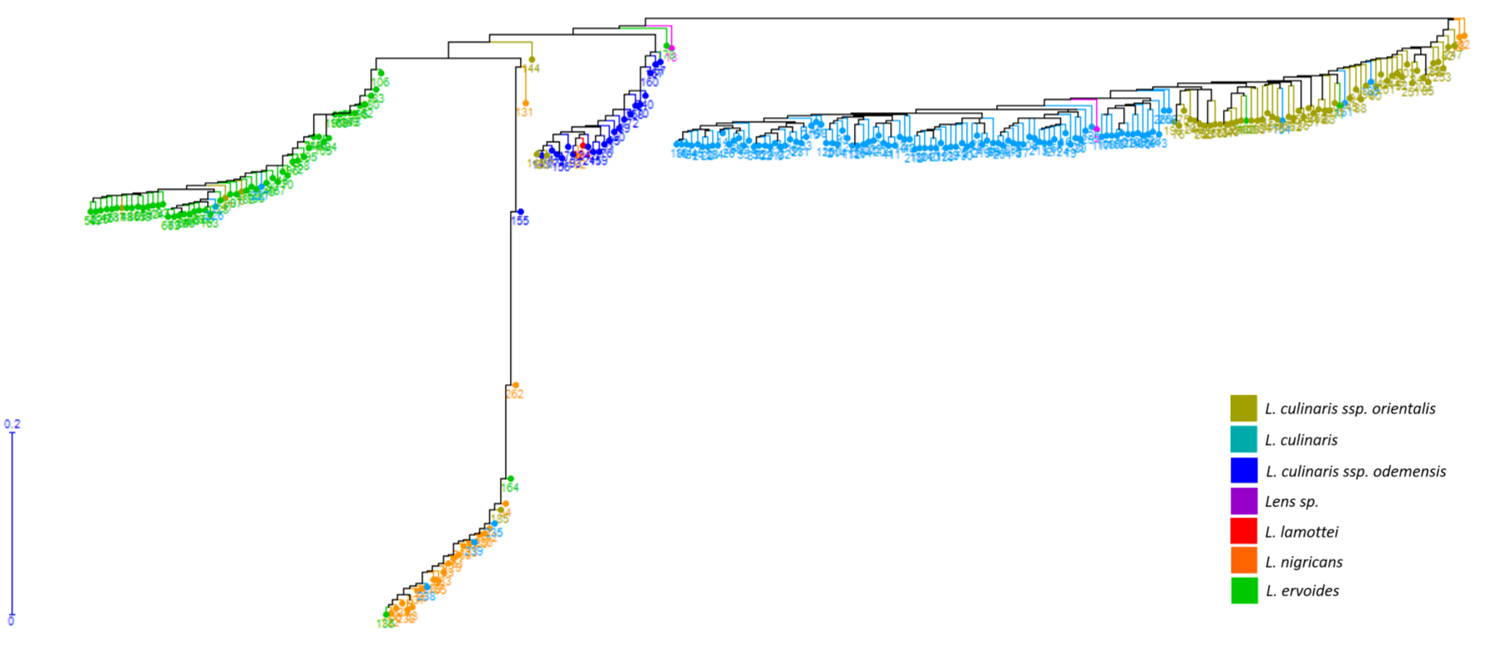

Supplement: FIGURE S1 — Unweighted neighbor joining dendrogram generated from all Lens species based on genetic distance calculation from StAMPP in R. Of the L. culinaris set, only a proportion of key accessions is included. [file Data_Sheet_1.ZIP › Supplementary files/Figure S1.tif]

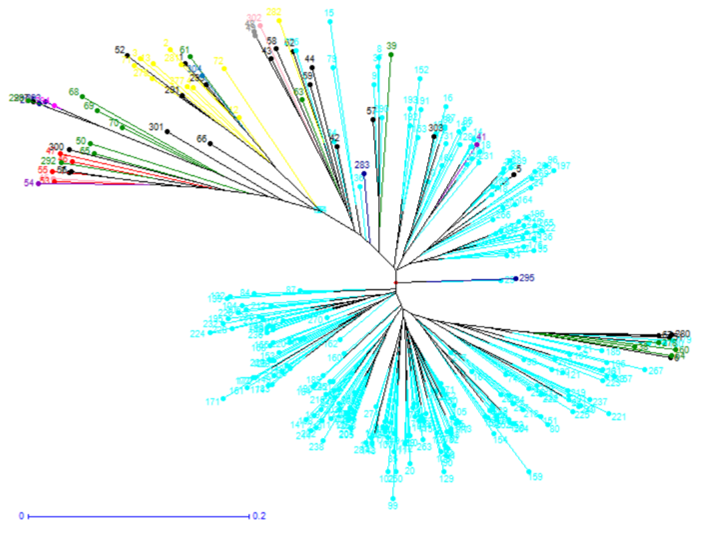

Supplement: FIGURE S1 — Unweighted neighbor joining dendrogram generated from all Lens species based on genetic distance calculation from StAMPP in R. Of the L. culinaris set, only a proportion of key accessions is included. [file Data_Sheet_1.ZIP › Supplementary files/Figure S2a.tif]

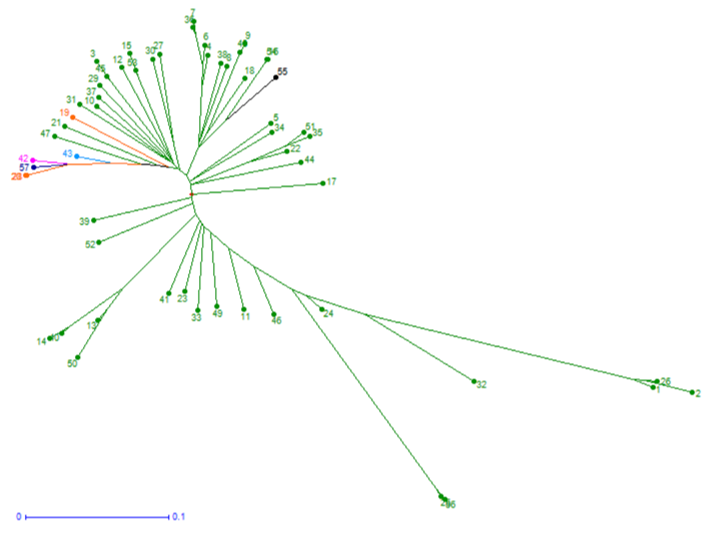

Supplement: FIGURE S1 — Unweighted neighbor joining dendrogram generated from all Lens species based on genetic distance calculation from StAMPP in R. Of the L. culinaris set, only a proportion of key accessions is included. [file Data_Sheet_1.ZIP › Supplementary files/Figure S2b.tif]

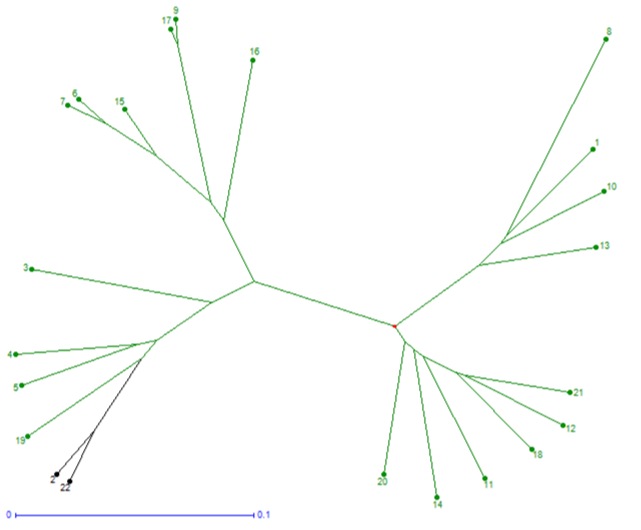

Supplement: FIGURE S1 — Unweighted neighbor joining dendrogram generated from all Lens species based on genetic distance calculation from StAMPP in R. Of the L. culinaris set, only a proportion of key accessions is included. [file Data_Sheet_1.ZIP › Supplementary files/Figure S2c.tif]

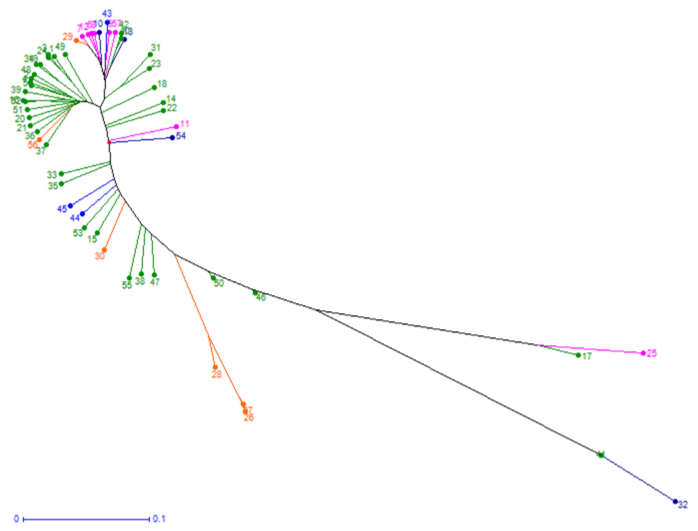

Supplement: FIGURE S1 — Unweighted neighbor joining dendrogram generated from all Lens species based on genetic distance calculation from StAMPP in R. Of the L. culinaris set, only a proportion of key accessions is included. [file Data_Sheet_1.ZIP › Supplementary files/Figure S2d.tif]

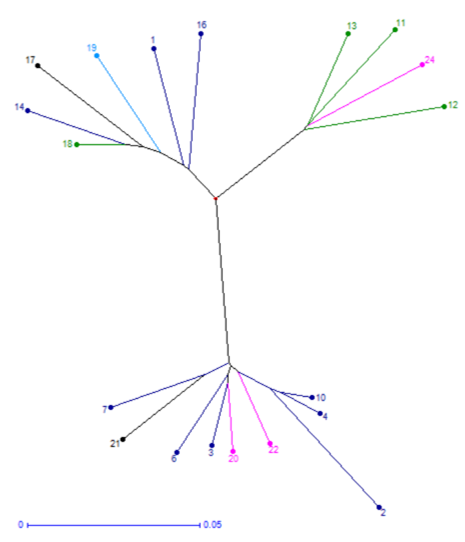

Supplement: FIGURE S1 — Unweighted neighbor joining dendrogram generated from all Lens species based on genetic distance calculation from StAMPP in R. Of the L. culinaris set, only a proportion of key accessions is included. [file Data_Sheet_1.ZIP › Supplementary files/Figure S2e.tif]

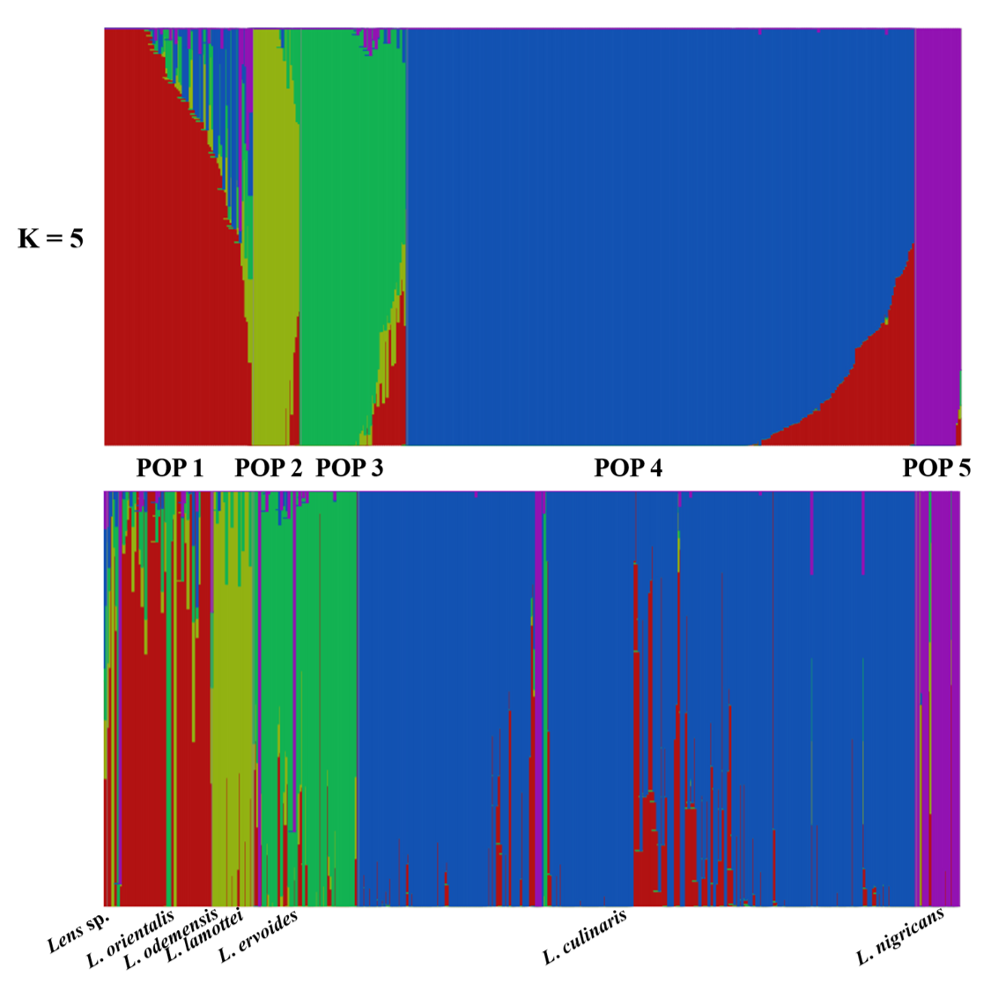

Supplement: FIGURE S1 — Unweighted neighbor joining dendrogram generated from all Lens species based on genetic distance calculation from StAMPP in R. Of the L. culinaris set, only a proportion of key accessions is included. [file Data_Sheet_1.ZIP › Supplementary files/Figure S3.tif]

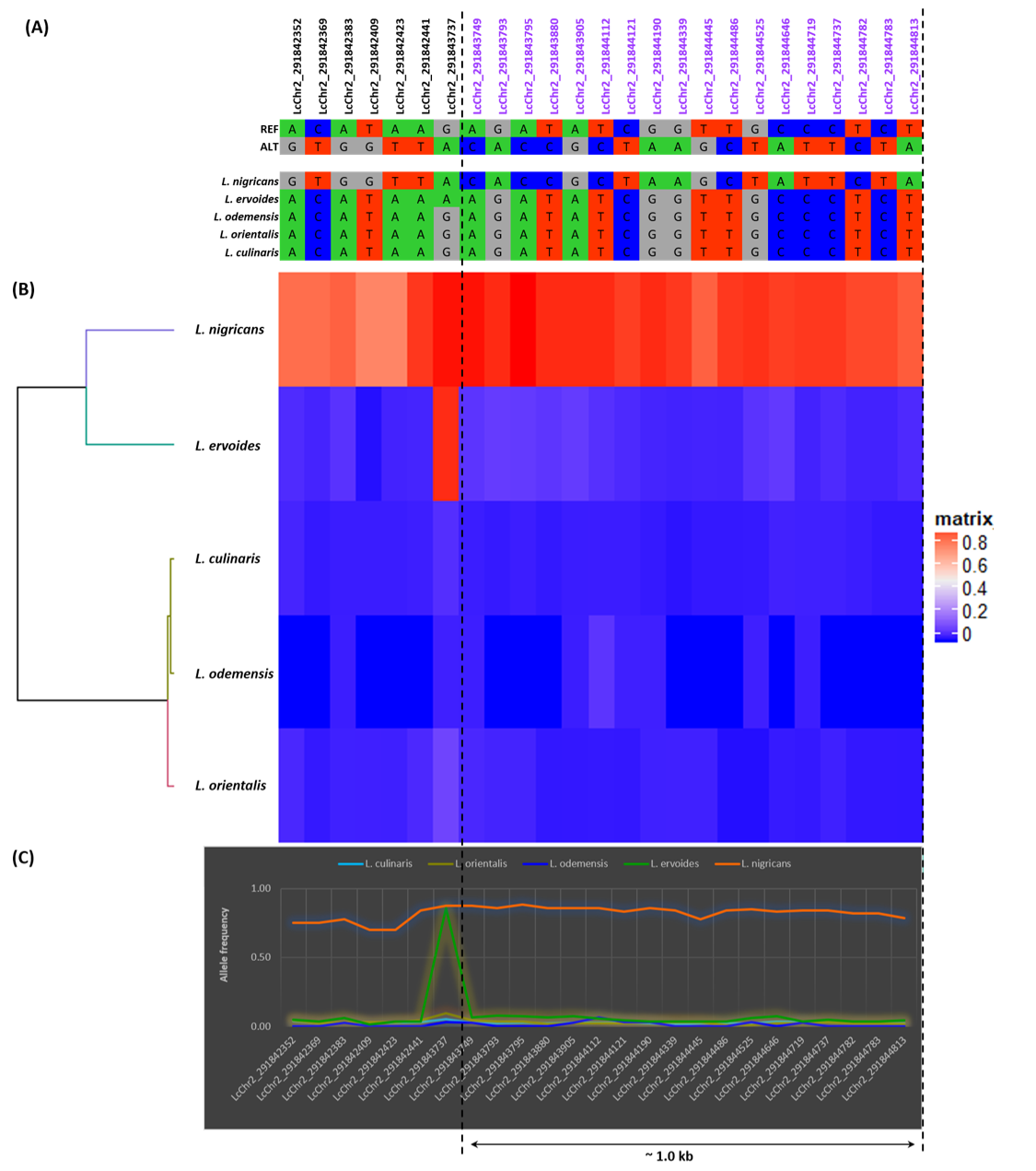

Supplement: FIGURE S1 — Unweighted neighbor joining dendrogram generated from all Lens species based on genetic distance calculation from StAMPP in R. Of the L. culinaris set, only a proportion of key accessions is included. [file Data_Sheet_1.ZIP › Supplementary files/Figure S4a.tif]

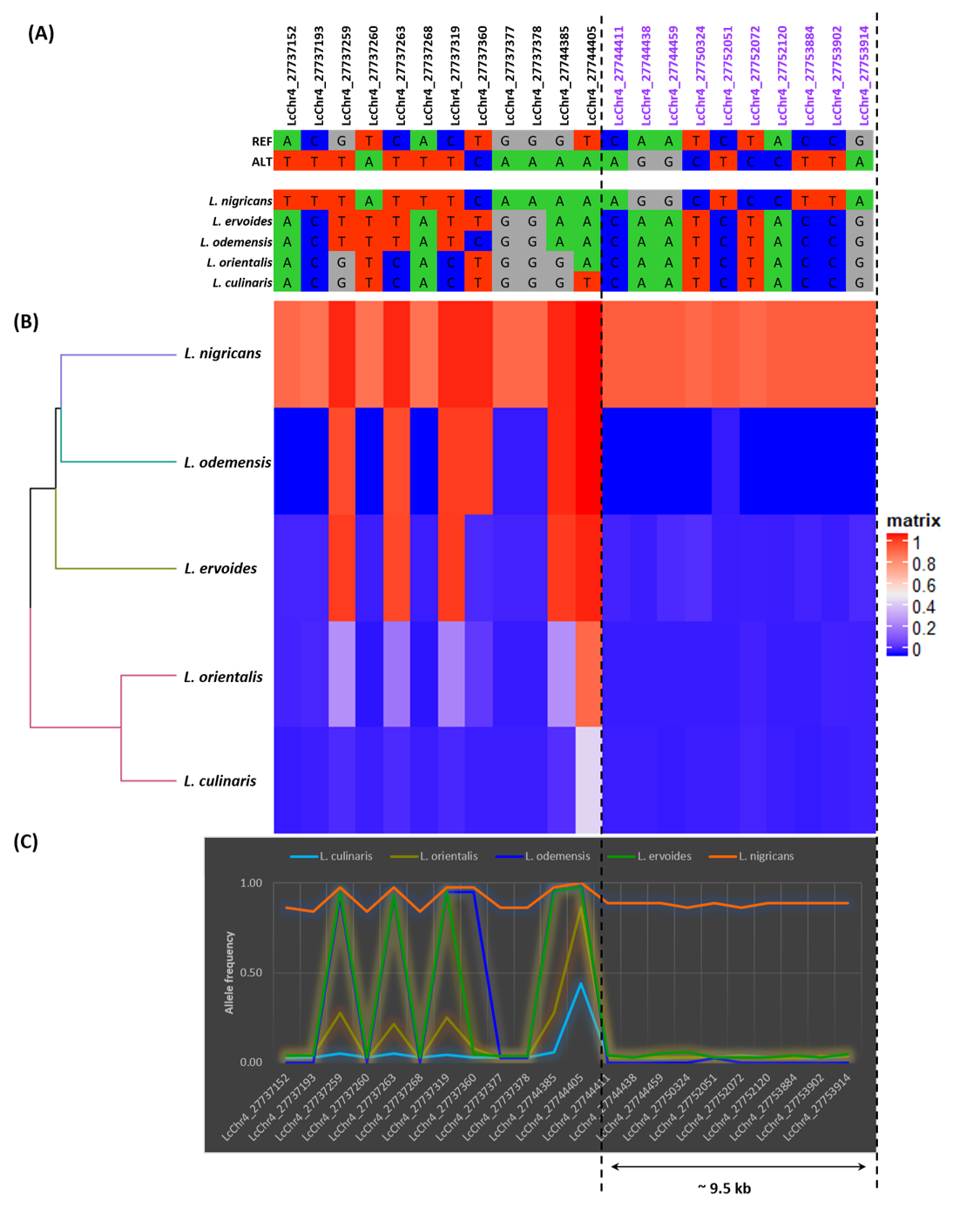

Supplement: FIGURE S1 — Unweighted neighbor joining dendrogram generated from all Lens species based on genetic distance calculation from StAMPP in R. Of the L. culinaris set, only a proportion of key accessions is included. [file Data_Sheet_1.ZIP › Supplementary files/Figure S4b.tif]

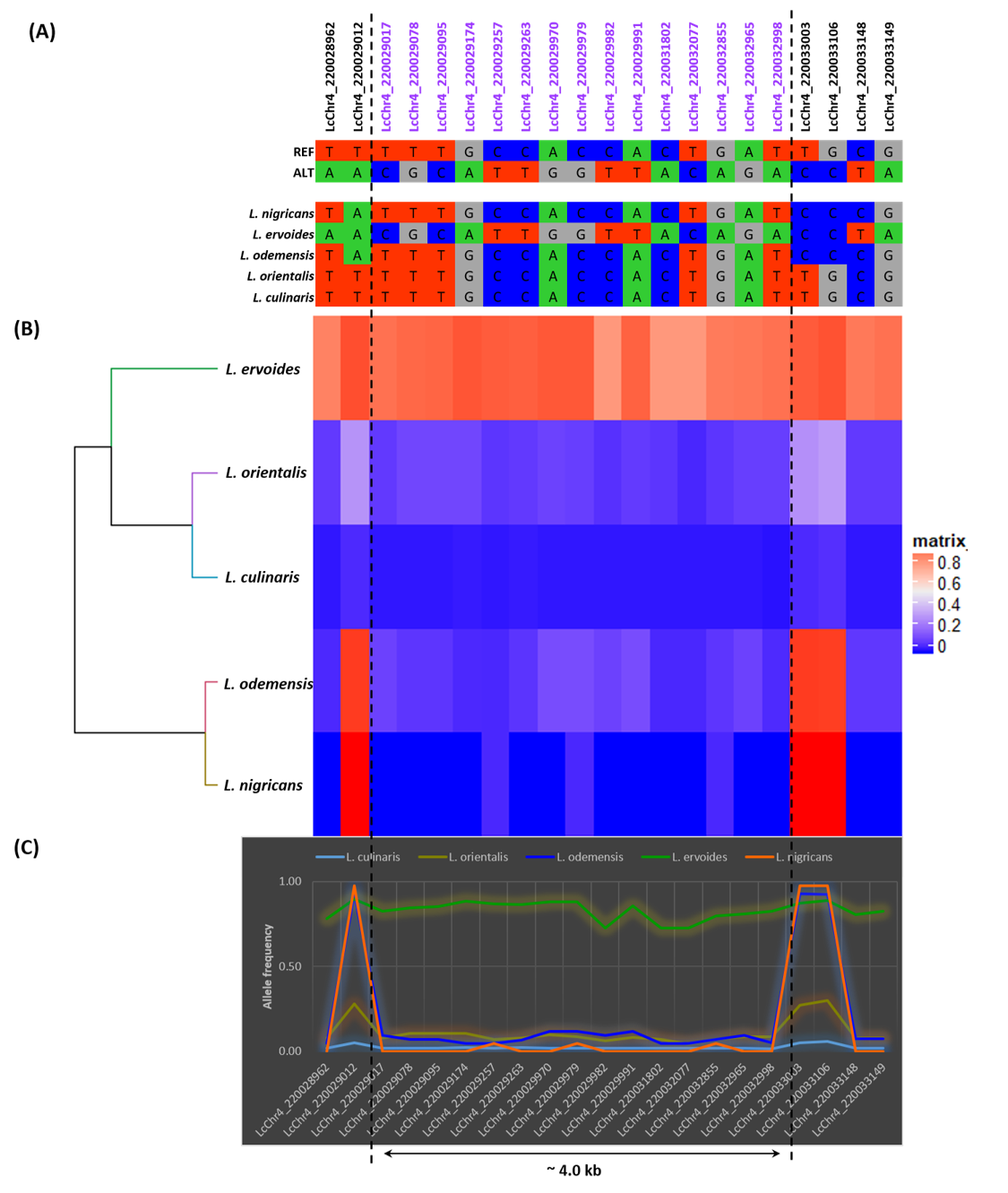

Supplement: FIGURE S1 — Unweighted neighbor joining dendrogram generated from all Lens species based on genetic distance calculation from StAMPP in R. Of the L. culinaris set, only a proportion of key accessions is included. [file Data_Sheet_1.ZIP › Supplementary files/Figure S4c.tif]

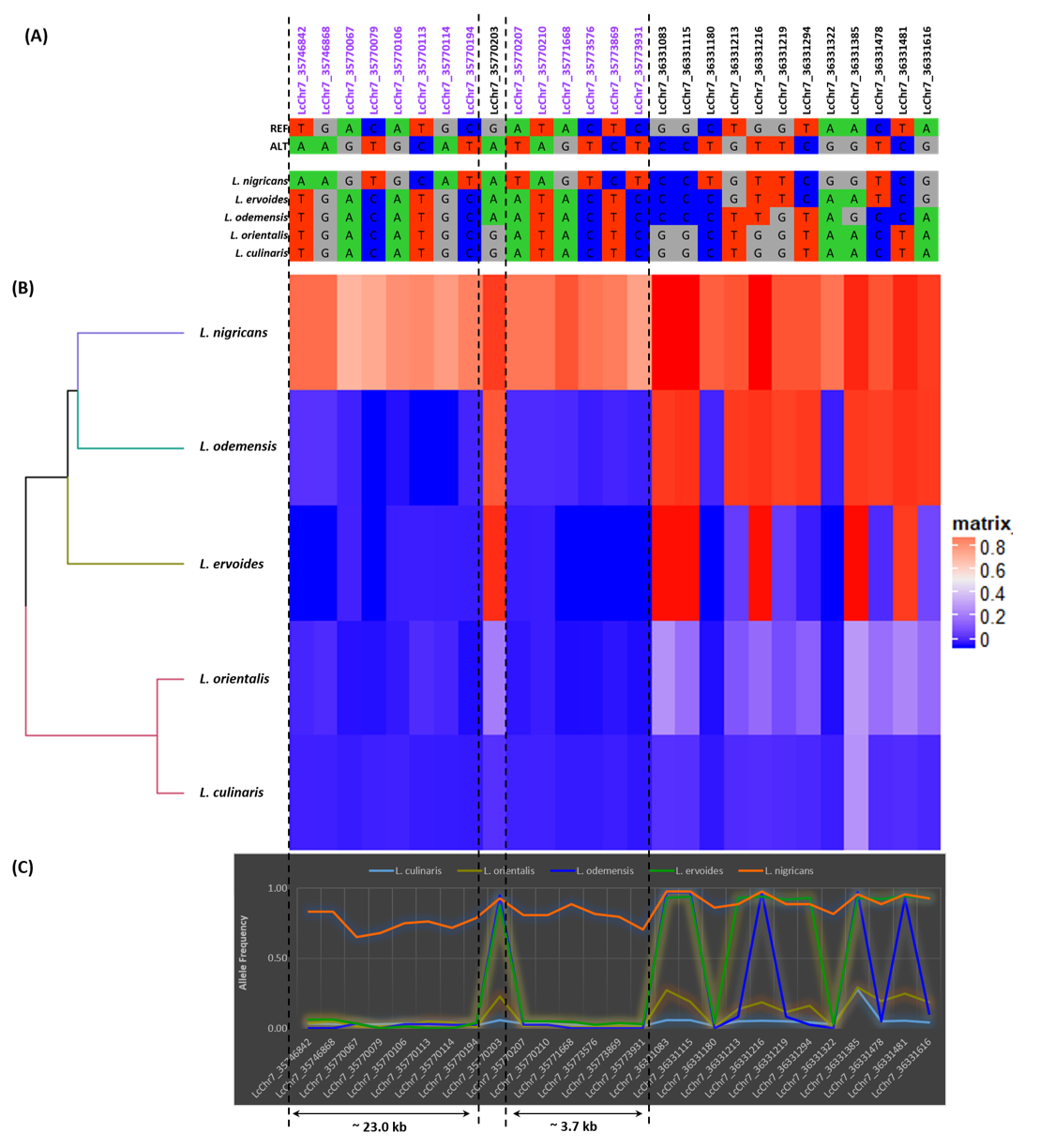

Supplement: FIGURE S1 — Unweighted neighbor joining dendrogram generated from all Lens species based on genetic distance calculation from StAMPP in R. Of the L. culinaris set, only a proportion of key accessions is included. [file Data_Sheet_1.ZIP › Supplementary files/Figure S4d.tif]
